# 747. Association of *Clostridiodes difficile* Infection Incidence With Renewed Vigor in Infection Prevention Practices With the Onset of the COVID-19 Pandemic

**DOI:** 10.1093/ofid/ofab466.944

**Published:** 2021-12-04

**Authors:** Ahmed A Khan, Sana Waqar

**Affiliations:** 1 Southern Illinois University School of Medicine, Springfield, IL; 2 Southern Illinois University, Springfield, IL

## Abstract

**Background:**

*Clostridioides difficile* is the leading cause of hospital associated infections. In 2017 it lead to an estimated 223,900 cases, 12,800 deaths and &1 billion in attributable healthcare costs.^[1]^ Judicious use of antibiotics and good hand hygiene practices form the cornerstone of prevention. During the COVID-19 pandemic there has been a focus on infection control practices such as hand hygiene, which would also lead to decreased incidence of other contagious infections such as *C. difficile* diarrhea.

**Methods:**

We looked at the incidence of *C. difficile* infection in a tertiary care hospital, 1 year before and 1 year after the start of the COVID-19 pandemic. We looked at the absolute number of hospital associated *C. difficile* infections and the rate per 1000 patient days. The testing methodology changed during the time of the study. Initially it included NAAT for *C. difficile*, however in March of 2020 the testing strategy included testing for GDH antigen and toxin A/B to differentiate between infection and asymptomatic colonization.

**Results:**

From January 1^st^ and December 31^st^ 2019 there were a total of 182 *C. difficile* infections with a rate of 1.29% per 1000 patient days. Between January 1^st^ and December 31^st^ 2020 there were a total of 51 *C. difficile* infections with a rate of 0.39% per 1000 patient days. There was an absolute risk reduction of 0.9% and relative risk reduction of 69.7%. Hand hygiene audits did not show a difference in adherence between the two periods, with a compliance rate of 98% for both.

**Conclusion:**

Our data suggests that there was a substantial reduction in *C. difficile* infection rate after widespread knowledge of COVID-19 and implementation of enhanced infection prevention strategies. These included frequent reminders of hand washing, gowning and social distancing to name some. This information was conveyed in the form of widely disseminated signs in highly visible areas, frequent reminders electronically and in person between staff and providers. There are limitations in our study, which include difficulty in longitudinally assessing the extent to which patient care providers adhered to infection prevention strategies and a change in testing strategy for *C. difficile* diagnosis during this time.

**Disclosures:**

**All Authors**: No reported disclosures

